# Vertical habitat preferences shape the fish gut microbiota in a shallow lake

**DOI:** 10.3389/fmicb.2024.1341303

**Published:** 2024-03-18

**Authors:** Bowei Zhang, Jiaman Xiao, Hongyan Liu, Dongdong Zhai, Ying Wang, Shujun Liu, Fei Xiong, Ming Xia

**Affiliations:** ^1^Hubei Engineering Research Center for Protection and Utilization of Special Biological Resources in the Hanjiang River Basin, School of Life Sciences, Jianghan University, Wuhan, China; ^2^Hubei Key Laboratory of Environmental and Health Effects of Persistent Toxic Substances, Jianghan University, Wuhan, China

**Keywords:** feeding habitat preference, trophic level, lake ecosystem, microbial community coalescence, fish gut microbiota

## Abstract

Understanding the interactions between fish gut microbiota and the aquatic environment is a key issue for understanding aquatic microorganisms. Environmental microorganisms enter fish intestines through feeding, and the amount of invasion varies due to different feeding habits. Traditional fish feeding habitat preferences are determined by fish morphology or behavior. However, little is known about how the feeding behavior of fish relative to the vertical structure in a shallow lake influences gut microbiota. In our study, we used nitrogen isotopes to measure the trophic levels of fish. Then high-throughput sequencing was used to describe the composition of environmental microbiota and fish gut microbiota, and FEAST (fast expectation-maximization for microbial source tracking) method was used to trace the source of fish gut microbiota. We investigated the microbial diversity of fish guts and their habitats in Lake Sanjiao and verified that the sediments indeed played an important role in the assembly of fish gut microbiota. Then, the FEAST analysis indicated that microbiota in water and sediments acted as the primary sources in half of the fish gut microbiota respectively. Furthermore, we classified the vertical habitat preferences using microbial data and significant differences in both composition and function of fish gut microbiota were observed between groups with distinct habitat preferences. The performance of supervised and unsupervised machine learning in classifying fish gut microbiota by habitat preferences actually exceeded classification by fish species taxonomy and fish trophic level. Finally, we described the stability of fish co-occurrence networks with different habitat preferences. Interestingly, the co-occurrence network seemed more stable in pelagic fish than in benthic fish. Our results show that the preferences of fish in the vertical structure of habitat was the main factor affecting their gut microbiota. We advocated the use of microbial interactions between fish gut and their surrounding environment to reflect fish preferences in vertical habitat structure. This approach not only offers a novel perspective for understanding the interactions between fish gut microbiota and environmental factors, but also provides new methods and ideas for studying fish habitat selection in aquatic ecosystems.

## 1 Introduction

Microorganisms are indispensable components of terrestrial life and can virtually coexist with any living organisms. Bacteria play an essential role in lake ecosystems by affecting food webs (Segovia et al., 2015), water quality (de Anda et al., 2019), and biogeochemical cycles (Newton et al., 2011). Lake pelagic bacterial communities exhibit strong seasonality in both abundance and composition, mostly driven by shifts in water temperature and inputs of allochthonous matter. In contrast, benthic bacterial communities appear to be more stable and less affected by seasonal shifts (Zhong et al., 2022). Meanwhile, fish, which were important parts in lake ecosystems, show discrepant behavior among different water layers (Endo and Watanabe, 2020; St. John et al., 2022). To the best of our knowledge, there have been no systematic studies of how water and sediments microbial communities in lakes affect the structure and function of gut microbes in fish with pelagic or benthic habitat preferences.

Fish gut microbiota play an important role in ecosystem microbial diversity (Xiao et al., 2021), affecting various aspects of the host, such as metabolism, feeding behavior, and immune response (Cresci and Bawden, 2015). Similar to mammals, the gut microbiota of fish can also be considered an important organ responsible for some key physiological functions that contribute to the health maintenance of the host (Bi et al., 2021). However, the mechanism governing aquatic fish gut microbiomes may be utterly different from those of terrestrial mammals (De Schryver and Vadstein, 2014). Previous studies have classified the factors that influence the composition of fish gut microbes into three categories: host factors, habitat factors, and dietary factors, and verified that these factors shaped the fish gut microbes, respectively, (Wong and Rawls, 2012; Chen et al., 2022). However, the primary determinant governing the composition of gut microbiota remains a subject of controversy. Recent research has shown that the composition of fish gut microbiota is closely linked to the host factors, such as: host genetics (Li et al., 2014), immunology (Stagaman et al., 2017), physiology (McDonald et al., 2012), development (Xiao et al., 2021) and ecology (Bolnick et al., 2014). These suggest that host-associated factors play a crucial role in shaping the fish gut microbiota. However, other studies have highlighted the importance of environmental factors in determining the fish gut microbiome (Sullam et al., 2012; Eichmiller et al., 2016; Kim et al., 2021). So far, the mechanism underlying fish-microbiota-environment relationships remains controversial (Xiao et al., 2021).

More recently, the importance of habitat in the assembly of fish gut microbes is being elucidated (Kim et al., 2021). In the wild condition, the interaction between fish gut microorganisms and environmental microbes have been gradually clarified by introducing-then-filtering framework (Yang et al., 2022). In adult fish, the microbes in gut contents are dominated by large numbers of transient microorganisms mostly derived from the water body (Le and Wang, 2020), and whose assembly is mainly driven by neutral introducing processes from the environment (Wong and Rawls, 2012; Heys et al., 2020; Le and Wang, 2020). In contrast, sediments, which always exhibit higher microbial diversity but lower transfer than water, have been found to significantly affect fish gut microbial composition by ingestion (Duan et al., 2021). However, the relative contributions of microbes in water or sediments to the assembly of fish gut microbial composition remain to be demonstrated.

Lake Sanjiao, an extensive fish culture lake, is connected to the Yangtze River as an important aquaculture place in Wuhan. The entire lake is eutrophic (Xia et al., 2022). Extensive aquaculture is characterized by the fact that the growth of fish and the production of the population depend exclusively (or mainly) on the local prey resources in the water (Oddsson, 2020), so that fish need to make full use of the resources in the water. Strong interactions between the water column and underlying sediments can influence biological processes more heavily in shallow lakes than in deeper lakes (Herb and Stefan, 2005). However, there have been few detailed interpretations of what the differences are and how they arrive among fish gut microbiota.

Here, we studied the microbiota in fish guts and the living environment in Lake Sanjiao, with the aim of addressing these longstanding questions about the gut microbiota: (1) Are there differences within the gut microbiota of fish in the lake ecosystem? (2) If the differences exist, what are the most important factors affecting fish gut microbes’ communities? (3) How do microorganisms in water and sediments affect fish gut microbes’ communities? To solve these problems, we collected 28 fish samples and 3 pairs of water and sediments samples from Lake Sanjiao and used high-throughput sequencing technology and stable isotope labels (SIL) to characterize factors affecting fish including host taxonomy, habitat microbiota and trophic level. We performed unsupervised machine learning first to cluster fish gut microbes. Supervised machine learning and traditional statistical methods were then used to verify the major factors affecting the gut microbial structure of fish. Our results provided the foundation for future studies of gut microbiota in fish or other aquatic animals.

## 2 Materials and methods

### 2.1 Sampling procedures

In March 2022, a total of 5 liters (L) of water and 500 grams (g) of sediments were collected at each sampling site across three designated sites within Lake Sanjiao using a 1/16 mud trap and a 5 L barrel water collector (Supplementary Table S1 and Supplementary Figure S1). Water and sediments at each site were sampled in three replicates. Fish were caught by sinking experimental gill nets. Using traditional taxonomic identification, we divided the collected fish into 7 species and selected 4 adults for each species (Supplementary Table S2). The 3 water samples and 3 sediments samples at each site were mixed separately and frozen in an on-board refrigerator set at-20°C and brought back to our laboratory. A total of 500 milliliters (mL) water sample in each site was filtrated with 0.22 μm filter membrane, and the filtered membrane was put into a 50 mL centrifuge tube, while the remaining water sample was used to measure the physicochemical properties. The fish were dissected with sterile scissors, and the hindguts were removed for the remainder of the study. The contents were transferred into a 50 mL centrifuge tube with sterile tweezers, and the gut microbial samples and environmental samples were stored in a −80°C freezer until DNA extraction.

### 2.2 DNA extraction

DNA was extracted from all samples (intestine, water, and sediments) using a bacterial DNA kit [TGuide S96 Soil/fecal Genome DNA extraction kit by magnetic bead method, Tiangen Biochemical Technology (Beijing) Co., Ltd.]. PCR amplification was performed based on the detection results. To perform 16S rRNA gene amplification analysis, primers 338F (ACTCCTACGGGAGGCAGCA) and 806R (GGACTACHVGGGTWTCTAAT) were used to amplify the V3–V4 hypervariable region of the 16S rRNA gene. The amplification procedure was run as set: predenaturation at 95°C (5 min), then denaturation at 95°C (30 s), annealing at 50°C (30 s), and extension at 72°C (40 s) for 25 cycles, culminating in elongation at 72°C (7 min). The amplified products were added to the magnetic beads and mixed at a 1:1 ratio for elution. After using the purified products, they were purified again by the Solexa PCR system. The procedure was run according to the setting: predenaturation at 98°C (30 s), then denaturation at 98°C (10 s), annealing at 65°C (30 s), and extension at 72°C (30 s) for 10 cycles, finally elongation at 72°C (5 min). Amplicons were extracted from 1.8% agarose gel and purified according to manufacturer’s instructions using Monarch DNA Gel Recovery Kit. Purified PCR products from each group were collected at equimolar concentrations. The platform and the instrument used for the sequencing was Illumina Novaseq6000 (Illumina, San Diego, CA, United States) in accordance with standard protocol of Biomarker Technologies, Inc. (Beijing, China) and the length of reads was PE250.

### 2.3 Sequence analysis

Trimmomatic (version 0.33) was used to filter the quality of the original data, and then Cutadapt (version 1.9.1) was used to identify and remove primer sequences. Subsequently, USEARCH (version 10) was used to splice the double-ended reads and remove the chimera (UCHIME, version 8.1) to obtain high-quality sequences for subsequent analysis. The DADA2 method in QIIME2 (version 2020.6) was used to de-noise the data after quality control. By default, 0.005% of all sequences were used as the threshold to filter ASVs. Representative sequences after denoising were annotated using KRAKEN on the galaxy platform.

### 2.4 Measurement of fish trophic level

The white muscles of the back of collected fish were sampled 2–3 g, rinsed with copper ion water, continuously dried at 60°C to a constant weight, and then preserved. All samples were analyzed for nitrogen isotope ratios using a Flash EA1112 HT element analyzer and DELTA V Advantage isotope ratio mass spectrometer from Thermo Fisher Scientific, United States. The standard material for nitrogen stable isotope analysis is standard atmospheric nitrogen (N_2_). In the experiment, 1 standard sample was added to every 5 samples, and 1–2 samples were selected by the machine for repeated determination in every 10 samples. The analysis accuracy was ±0.2‰.

The formula for calculating the stable isotope ratio of nitrogen (D’Ambra et al., 2014) is as follows:


$$\delta^{15}N=\left(R_{s}-R_{st}\right)/R_{st}\times 1000$$

where, R_s_ represents the ratio of heavy isotope to light isotope of the sample (^15^N/^14^N); R_st_ is the standard atmospheric nitrogen isotope ratio.

The formula for calculating the trophic level is as follows:


$$\mathrm{TL}=\left(\delta^{15}N_{\mathrm{consumer}}-\delta^{15}N_{\mathrm{reference}\;\mathrm{organism}}\right)/\Delta \delta^{15}N+\lambda$$

where, TL is the trophic level of the consumer, δ^15^N_consumer_ is the nitrogen stable isotope ratio of the consumer, δ^15^N_reference organism_ is the nitrogen stable isotope ratio of the reference organism in the system, ∆δ^15^N is the nitrogen stable isotope enrichment degree between adjacent trophic levels, λ is the trophic level of the reference organism, *λ* = 1 when the primary producer, *λ* = 2 for primary consumers. In this study, the nitrogen stable isotope enrichment degree (∆δ^15^N) between adjacent trophic levels was 3.4‰, and primary consumer *Bellamya aeruginosa* were selected as the reference organism (Supplementary Table S3).

### 2.5 Extent of coalescence between microbial communities

**Figure 1:**
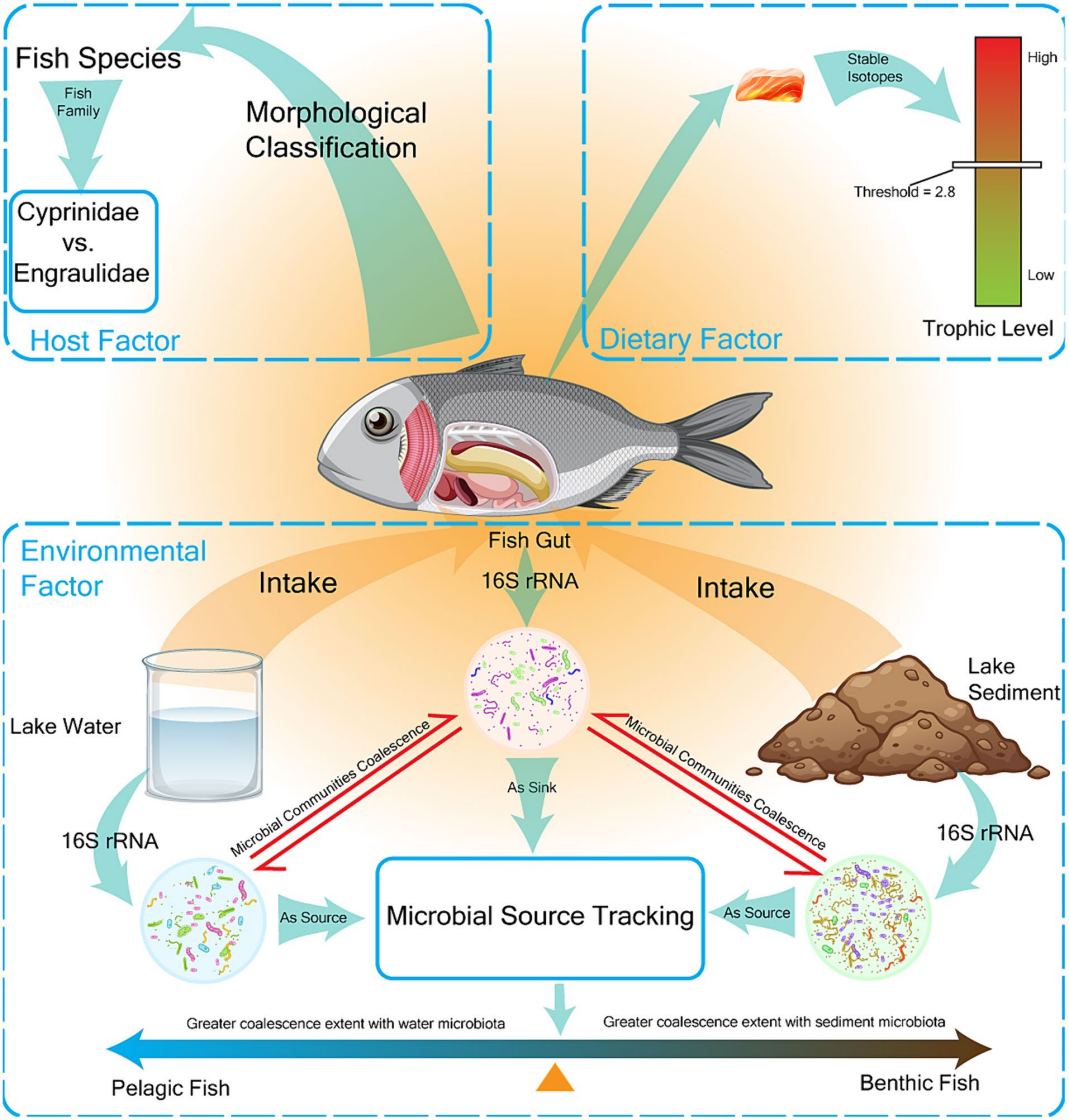
Main factors affecting the composition of the gut microbiome in fish were measured. The factors affecting gut microbiota composition were classified into three categories: host factors, dietary factors, and environmental factors, which were expressed as fish species, fish trophic level, and fish habitat preference, respectively. Fish were classified into two families (*Cyprinidae* vs. *Engraulidae*) and seven species (AN *Aristichthys nobilis*, CA *Carassius auratus*, CB *Coilia brachygnathus*, CD *Culter dabryi*, HL *Hemiculter leucisculus*, PS *Pseudobrama simoni*, TS *Toxabramis swinhonis*) by morphological classification. Fish trophic level was determined by stable isotope determination with a threshold of 2.8. Fish habitat preference was determined by the extent of coalescence between gut microbes and environmental microbes, and fish were divided into pelagic and benthic fish.

The environmental factors that affect the composition of fish gut microbiota include biotic processes (the invasion of habitat microbiota) and abiotic factors (habitat physical and chemical properties) (Chen et al., 2022). Microbial community coalescence refers to the mixing of microbial communities and the merging of their surrounding environments, which is an important biological indicator of environmental factors (Gao et al., 2021). In this study, microbial community coalescence was used to quantify the influence of environmental microbes on the invasion of fish gut microbiota. We used fast expectation-maximization for microbial source tracking (FEAST) (Shenhav et al., 2019) to trace the origins of fish gut microbes, using fish gut microbial communities as sinks and water/sediments microbial communities as sources. Results from FEAST were used to represent the extent of microbial community coalescence (Gao et al., 2021) (Figure 1).

### 2.6 Data analysis

We calculated α-diversity of samples, the Bray–Curtis distance matrix and the ANOSIM coefficient between different groups using the “vegan” package in R and obtained the false discovery rate (FDR) by correcting the *p*-value of ANOSIM through Benjamini–Hochberg method. The significance of each ANOSIM parameter was permuted 999 times. The “cluster” (Mächler et al., 2012) and “clusterSim” (Dudek and Walesiak, 2020) packages in R were used for unsupervised machine learning clustering of fish guts microbiota. To determine the optimal number of clusters for evaluating clustering cohesion with various metadata, the Calinski–Harabasz index and profile scores for each cluster generated by PAM clustering were calculated. We used the R packages “randomForest” (Breiman, 2001) and “pROC” (Robin et al., 2011) to conduct supervised machine learning on the fish guts microbiota based on different influencing factors and evaluated the effect of each grouping according to the area under the curve (AUC) of each classifier’s ROC curve. Using LEfSe and PICRUSt2 (Douglas et al., 2020) via the Galaxy server further substantiated the dissimilarities in microbial species and their respective functions among the groups. The iNAP (Feng et al., 2022) platform was used to construct intergroup microbial networks based on spearman correlation coefficients, and the attributes of nodes, edges, and modules between networks were analyzed. In Cytoscape visualization, ASVs were used as nodes in the network diagram, and the connected edges were spearman correlation coefficients. Co-occurrence network stability was calculated by the software “fastnc2” in the conda environment (Wu et al., 2021). According to “fastnc2” and its supporting references, adjacent matrices (0 and 1 matrices, with 0 representing no edges between indicators and 1 representing edges between indicators) were extracted from the networks. Then nodes were randomly deleted in succession, and the natural connectivity of the remaining matrices were calculated. Each random deletion procedure was iterated 1,000 times and the results of 1,000 calculations were averaged. This process can be considered as a method to simulate the random disappearance of species using random sampling to grab nodes randomly and delete them. By making a linear regression between the calculation results and the proportion of deleted nodes and extracting the slope value, the slope value between different networks was compared to reflect the stability of the network. The network with a higher decline slope of natural connectivity value has worse network stability.

## 3 Results

### 3.1 Microbiota composition of the fish gut and their habitats in Lake Sanjiao

We analyzed the gut microbiota composition of 28 fish and the surrounding water microbiota and sedimental microbiota at three sites in Lake Sanjiao (Supplementary Table S1 and Supplementary Figure S1). The collected fish were divided into 7 kinds according to species classification (AN *Aristichthys nobilis*, CA *Carassius auratus*, CB *Coilia brachygnathus*, CD *Culter dabryi*, HL *Hemiculter leucisculus*, PS *Pseudobrama simoni*, TS *Toxabramis swinhonis*) (Supplementary Table S2). Overall, 3,200,011 pairs of raw sequences were found from 28 fish intestinal samples and 6 environmental samples, and 3,195,093 clean reads were produced after double-ended sequence quality control and splice. The high-quality variants were denoised and a threshold of 5 parts per 100,000 was set for the 1,204 ASVs in the variants.

**Figure 2:**
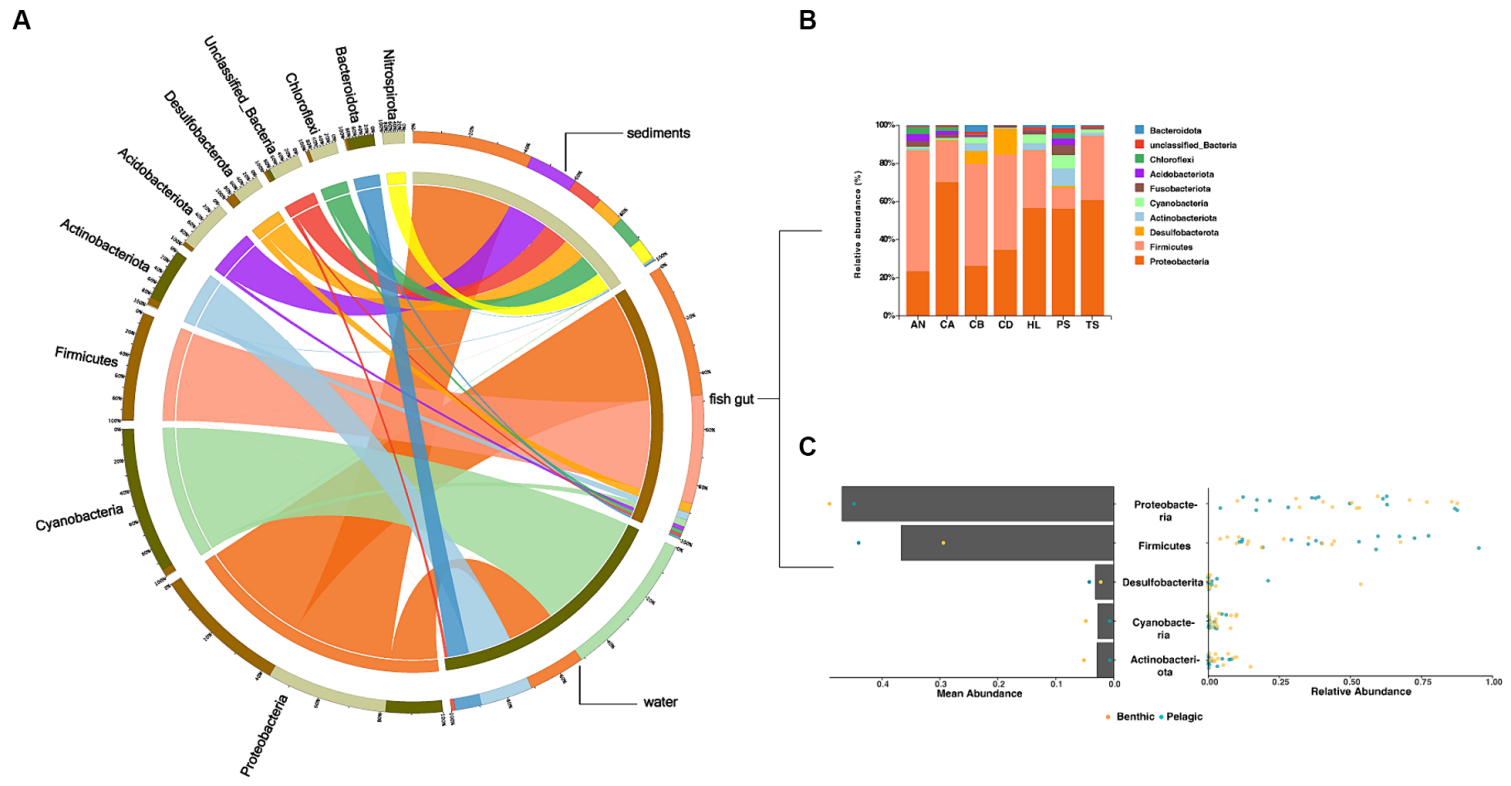
Overview of the data. **(A,B)** Circos chart and bar chart of the relative abundance of bacterial phyla (top 10) in the microbiota in (**A** all samples **B** fish samples). **(C)** Dot plot of the overall distribution of the relative abundance (right) and mean abundance (left) of taxa in total fish (bar) and pelagic fish or benthic fish (dot) at the bacterial phylum level.

The fish gut microbiota included 22 phyla, including 2 dominant phyla (Proteobacteria and Firmicutes) (Figures 2A,B). Bacteria in water and sediments contained 15 and 17 phyla, respectively. Cyanobacteria were the predominant group in water samples, while Proteobacteria dominated in sediments samples. All dominant phyla accounted for more than 30% of all sequence reads (Figures 2A,C).

We then performed a comparative analysis of fish gut and environmental microbiota in α-diversity by Shannon index, results showed that the environmental microbiota exhibited significantly higher richness than those in the fish gut. Furthermore, the sediments showed the highest microbial α-diversity among the total samples (Supplementary Figure S2).

### 3.2 Contribution of different habitats to fish gut microbes in Lake Sanjiao

**Figure 3:**
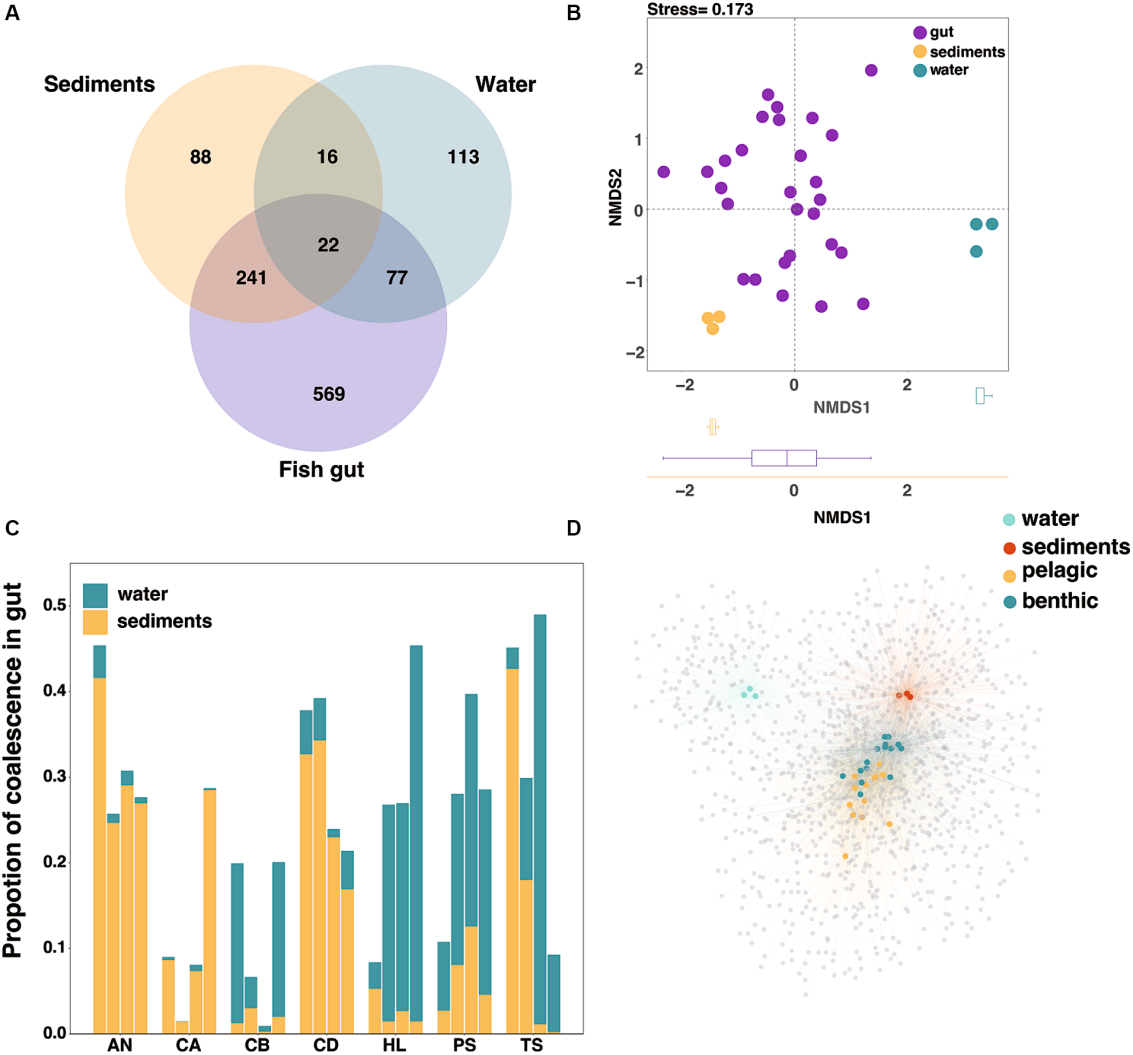
Interactions of microbes in water and sediments with gut microbes at the ASV level. **(A)** Venn diagram of the core microorganisms in the gut microbiota, water microbiota and sediments microbiota. **(B)** The NMDS analysis based on the Bray–Curtis distance to characterize the similarity of water microbiota, sediments microbiota and fish gut microbiota. **(C)** The proportion of coalescence between fish gut microbial communities and habitat (water/sediments) microbial communities, calculating by FEAST. **(D)** The network analysis based on species abundance, with colored nodes representing gut and environmental samples and gray nodes representing ASVs within species. The blue and yellow nodes represented fish samples with different habitat preferences, and the edge width and transparency represented the ASV abundance in the sample.

To evaluate the contribution of the environmental factors to the fish gut microbiota diversity, we analyzed the relationship between microbiota in environment and fish gut at the ASV level in Lake Sanjiao. The results showed that the majority of ASVs in the sediments were shared with fish guts (71.7%), while less than half of the ASVs in the water were shared with fish guts (43.4%) (Figure 3A). NMDS analysis based on the Bray–Curtis distance verified that the fish gut microbiota was more similar to that of sediments than to that of water (Figure 3B). All above suggested that the water and sediments habitat may exchange microbiota with fish gut in different degrees, and that the effect of sediments on fish cannot be ignored.

In the absence of differences in habitat physical and chemical properties (Supplementary Table S1), we used the environmental microbial invasion process to represent the influence of environmental factors on the fish gut microbial composition. In order to uncover the coalescence extent between fish gut microbiota and environmental microbiota, we then traced the source of fish gut microbes through fast expectation-maximization for microbial source tracking (FEAST), as previously described (Shenhav et al., 2019), and the relative contributions of different habitats varied among fish samples (Figure 3C). The FEAST results were used to represent the extent of environmental microbial invasion of the fish gut. Considering that fish have different intake of water and sediments under different water layers, the extent of microbial community coalescence can well reflect this intake relationship (Yang et al., 2022). Here we described the fish whose gut microbiota exhibited a greater coalescence extent with water microbiota as the pelagic feeding fish, and we identified the benthic feeding fish as those whose gut microbiota exhibited a greater coalescence extent with sediments microbiota.

We used a network-based approach to test whether gut microbial communities could be clustered by fish habitat at the ASV level. In agreement with the compositional differences noted above, the host nodes were more likely to connect to nodes of other hosts sharing the same habitat than to those from different habitats (Figure 3D). Furthermore, we found that pelagic fish had a significantly higher number of connections (i.e., degree) and higher betweenness centrality than those of benthic fish, while benthic fish had a higher neighborhood connectivity (Supplementary Table S4). These results suggested that intestinal microbial diversity in pelagic fish was greater than that in benthic fish, analogous to the results based on α-diversity estimates (Supplementary Figure S2).

### 3.3 Evaluating the key factors influencing intestinal microbiota of fish in lakes

It is known that environmental factors, host factors, and dietary factors jointly shape the fish gut microbiota (Chen et al., 2022). Host habitat has been confirmed to be the dominant determinant of gut microbiome in wild fish through large-scale surveys (Kim et al., 2021). However, it is still urgently needed to clarify how these factors affect the microbial composition of fish in specific local environment investigations in freshwater. In our study, the effects of environmental factors were represented by the fish’s preferences for different feeding habitats (pelagic/benthic), and the preferences were calculated by the coalescence extent of the fish gut microbiota and habitats microbiota. Host factors were represented by the taxonomy of the fish in lake and dietary factors were represented by the trophic levels of fish (Figure 1 and Supplementary Table S3).

**Figure 4:**
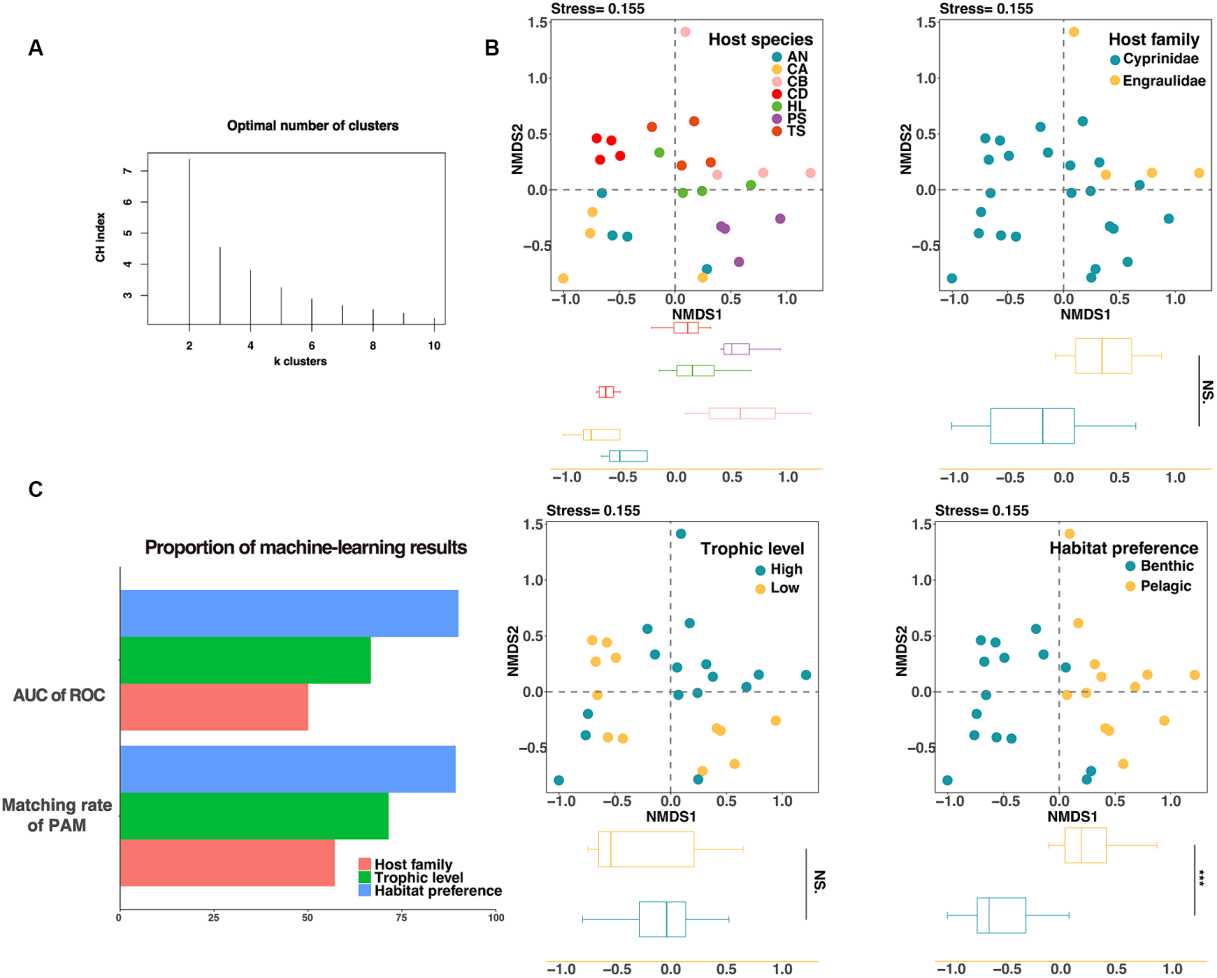
Evaluating the grouping of gut microorganisms. **(A)** The CH index was used to calculate the optimal number of clusters for gut microorganisms, and it was shown that *k* = 2 results in the best clustering of gut microorganisms. **(B)** The NMDS analysis based on Bray–Curtis distance showed the classification effect of fish intestinal microorganisms in different species (top left), different families (top right), different trophic levels (bottom left), and different habitat preferences (bottom right). On the NMDS1 axis, only habitat preferences showed significant differences (*p* < 0.001). **(C)** Unsupervised machine learning (PAM) and supervised machine learning (Random Forest) were used to evaluate the groups of fish intestinal microorganisms, and the fish habitat preferences score was the highest.

We used the Calinski–Harabasz index and the silhouette score to determine the optimal clustering number, and then used the partition around medoids clustering algorithm (PAM) to investigate the importance of habitat preferences, host taxonomy, and host trophic level. The PAM clustering result showed that the gut microbiota of fish could be clustered into two groups (Figure 4A), and the variation between groups were more consistent with differences between habitat preferences (pelagic vs. benthic) than those between host families (Cyprinidae vs. Engraulidae) or trophic levels (threshold = 2.8) (Supplementary Table S3 and Figure 4C). Among the three influencing factors, the habitat preferences had the highest proportion of correctly matched constituents with the results of PAM, indicating that habitat preferences were the primary determinant of fish gut microbiota. We additionally used analysis of similarities (ANOSIM) based on weighted unifrac distance to evaluate the significance of the effects of various candidate factors on the gut microbiota. Results showed that host trophic level (ANOSIM *R* = 0.020, *p* = 0.297) did not significantly distinguish fish gut in lakes, whereas host habitat preferences (ANOSIM *R* = 0.52, *p* < 0.001) and host taxonomies (species, ANOSIM *R* = 0.395, *p* < 0.001; family, ANOSIM *R* = 0.429, *p* = 0.004) significantly affected the microbial structure of the fish gut. It should be noted that the habitat preferences had the greatest ability to distinguish among samples (Supplementary Figure S3).

The supervised machine learning algorithm was then used to evaluate the three factors, and the area under the roc curve (AUC) was used to calculate the classification accuracy. The results showed that the ability of intestinal microbial communities of lake fish to distinguish between different habitats was higher than that of host taxonomy or trophic levels under the grouping by machine learning (Figure 3C).

### 3.4 Taxonomic differences between intestinal microbiota of pelagic and benthic fish

In order to verify the effect of habitat preferences on fish gut microbiota, we investigated differences in the composition of the gut microbiota with respect to the habitat preferences. In gut microbiota, 3 phyla were detected to be significantly different between pelagic fish and benthic fish (Supplementary Figure S4, threshold >4). The result showed that phylum Proteobacteria was enriched in benthic fish guts with a relatively high LDA score (LDA >4), while the phyla Actinobacteria and Bacteroidetes were significantly enriched in pelagic fish guts. Alphaproteobacteria in class level and *Clostridium botulinum* in species level both showed the highest score among the groups.

**Figure 5:**
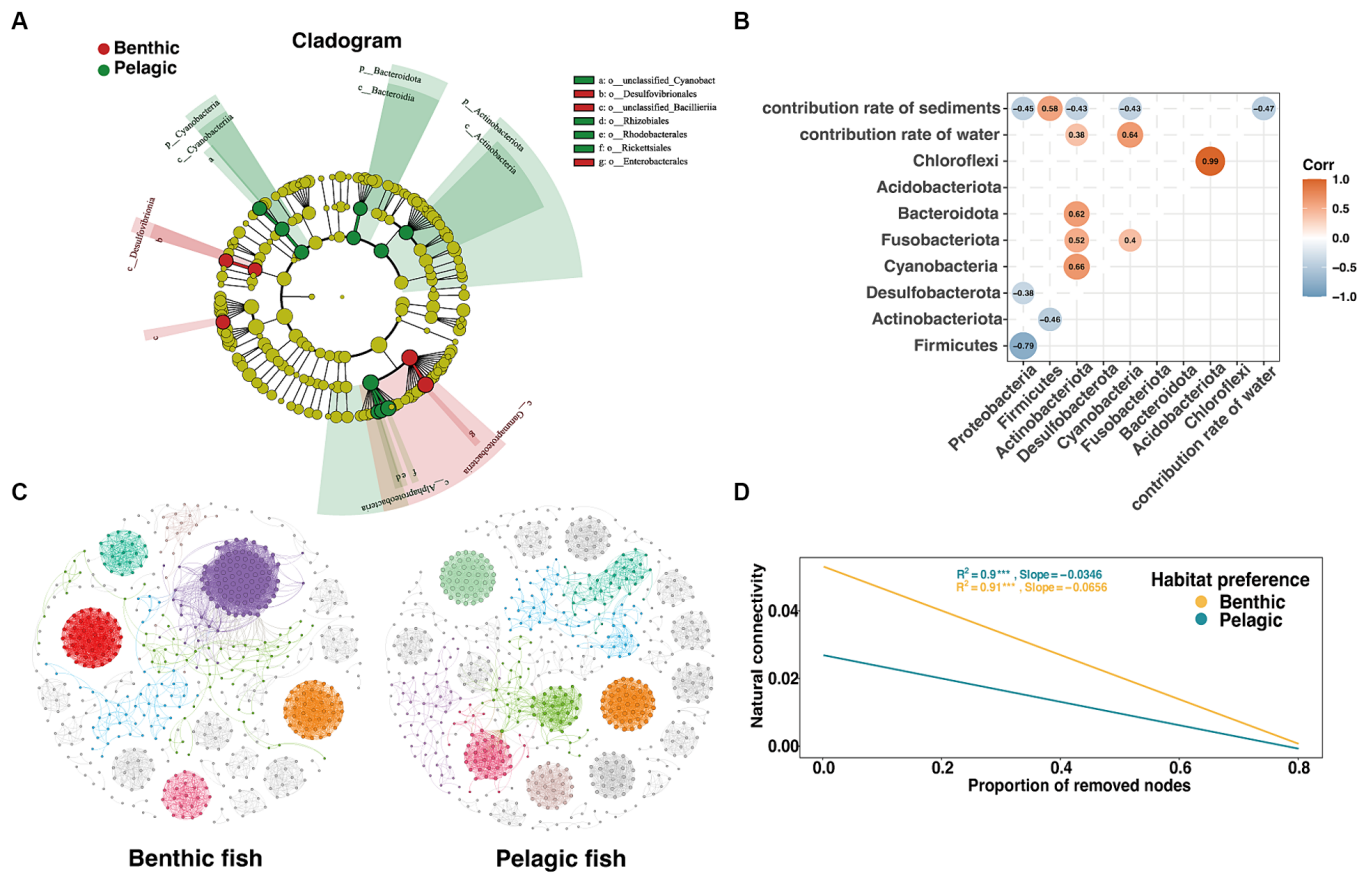
Analysis of intergroup differences in habitat preferences of intestinal microorganisms in fish. **(A)** Cladograms based on LDA scores with a threshold of 4 show the diversity of species from phylum to destination group. **(B)** Heatmap based on spearman correlation coefficient, only significant (*p* < 0.05) r values between microbial phyla and different indices are shown. **(C)** Benthic (left) and pelagic (right) fish gut microbiota co-occurrence network based on spearman correlation coefficient with the threshold of 0.8 through RMT calculation (Deng et al., 2012), different colors represent different modules in the network. **(D)** The trend line that natural connectivity decreased with the proportion of removed nodes in the two co-occurrence networks.

A heatmap was used to show the correlation between the phylum of fish gut microbiota and the coalescence extent of environmental microbiota using FEAST results (Figure 5B). A strong positive correlation was found between Acidobacteriota and Chloroflexi, while Proteobacteria showed a negative correlation with Firmicutes within the gut microbiota. An intriguing phenomenon was that although Proteobacteria were the most abundant phylum in sediments, the relative abundance of Proteobacteria appeared to decrease with the increasing relative contribution of sediments microbiota.

A co-occurrence network was used to characterize the gut microbial structure of fish based on spearman correlation coefficients (Figure 5C). In agreement with the network based on relative abundance (Figure 3D), we found that pelagic fish co-occurrence network had a significantly higher betweenness centrality than those of benthic fish co-occurrence network (Supplementary Table S5). Furthermore, with the random removal of the nodes, the pelagic fish gut microbiota network connectivity decreased more slowly, indicating that the network was more stable (Figure 5D).

### 3.5 Functional profiling of pelagic and benthic fish microbial communities

**Figure 6:**
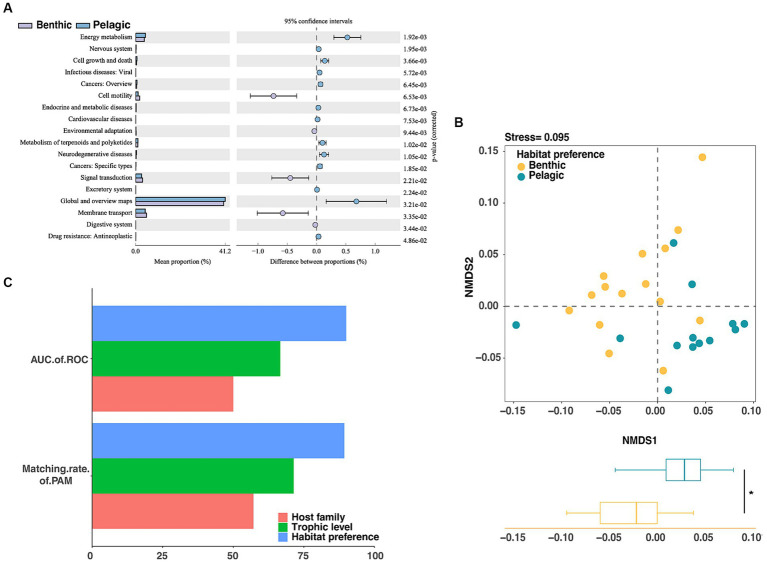
Assessment of gut microbiota grouping at functional level in fishes through **(A)** KEGG class 2 variation analysis of metabolic pathways. **(B)** The NMDS analysis showed significant differences in intestinal microbes of fish with different habitat preferences (*p* < 0.05). **(C)** Unsupervised machine learning (PAM) and supervised machine learning (Random Forest) were used to evaluate the groups of fish intestinal microorganisms based on functional profile, the fish habitat preferences score was the highest.

To figure out whether habitat preferences can affect the gut microbiota on functional level, we used the PICRUSt2 pipeline to make functional predictions for the groups of pelagic fish and benthic fish. Functional categories predicted from the 16S rRNA gene sequences were built based on the Kyoto Encyclopedia of Genes and Genomes (KEGG) ortholog groups (KOs) (Figure 6A). NMDS based on KOs predicted by PICRUSt2 illustrated that the host habitat preferences significantly affected the functional gene distribution (NMDS1, *p* < 0.05) (Figure 6B).

Significantly differential functions of fish gut microbiota between the two groups with different habitat preferences were performed by LEfSe analysis with the LDA score threshold of 3. Gene families in the following categories were enriched in pelagic fish: energy metabolism, cell growth and death, viral diseases, endocrine and metabolic diseases, metabolism of terpenoids and polyketides, neurodegenerative discases and antineoplastic resistance. By contrast, gene families in the following categories were enriched in benthic fish: cell motility, environmental adaptation, signal transduction, membrane transport and digestive system (Supplementary Figure S5).

We then used a machine learning approach to examine whether the functional profiles of the gut microbiota could be used to predict the environment or host taxonomy. The AUCs of ROC and matching rates of PAM calculated using the functional profiles showed better prediction accuracy for the habitat preferences than for other factors, consistent with the results of the random forest classifier and K-medium analysis based on microbial taxonomic profiles (Figure 6C).

## 4 Discussion

### 4.1 Fish gut microbiota can be the indicator for discerning the vertical structure of fish habitats

As a crucial component of lake ecosystems, fish possess remarkable mobility and are considered as the most evolutionarily successful vertebrates (Colston and Jackson, 2016), largely due to their gut microbiota (Clements et al., 2014; Ghanbari et al., 2015). Previous research has identified potential associations between fish gut microbes and environmental factors, primarily in terms of horizontal habitat structure (Le and Wang, 2020; Pan et al., 2023). Given that the vertical habitat preferences of fish are directly related to behaviors such as feeding, and fish feeding can introduce the invasion of environmental microorganisms (Yang et al., 2022). We suspected that vertical fish feeding can make a difference in gut microbial compositions through the invasion of environmental microorganisms.

The living environment of fish has been seen as the most important factor affecting the gut microbiota of fish in most current studies (Kim et al., 2021; Yang et al., 2022). Nearly all fish in our study were labeled as benthic according to FishBase (*Aristichthys nobilis*, *Carassius auratus*, *Culter dabryi*, *Hemiculter leucisculus*, *Pseudobrama simoni* and *Toxabramis swinhonis*). However, our analysis of fish gut microbial traceability data revealed that water and sediments, respectively, acted as the primary source for half of our samples (Figure 3B). Although the description of fish habitat in FishBase is broad, this rule is not perfectly applicable for all aquatic environments, as well as fish behavior is uncertain. Therefore, neither traditional fish ethology (i.e., DVM) nor fish morphology (i.e., FishBase) are not good indicators of fish habitat preferences. Here, we chose to use the intestinal microbiota to quantify the invasion of water and sediments microorganisms associated with feeding. We defined the fish whose gut microbiota come more from sediments than from water as benthic feeding fish, while others were considered as pelagic feeding fish (Supplementary Table S2).

The pelagic microbes were thought more directly affected by external episodic disturbances such as rainfall (Andersson et al., 2014), environmental influences (Rösel et al., 2012), storms and strong winds, solar radiation (Pérez and Sommaruga, 2007), and anthropogenic influences (Zhang et al., 2020). Similarly, pelagic fish in our study showed higher exogenous transients in their gut microbiota than benthic fish (Figure 5). For example, Cyanobacteria and Rhizobiales, enriched in the gut of pelagic fish (Figure 5C), were universally recognized as a component of the water microbiota in lakes (Huang et al., 2022). For benthic organisms, especially bottom feeders, sediments were major sources of bacteria that contribute to the formation of the gut microbiota (Zhang et al., 2021; Zhou et al., 2021). In addition, for benthic fish, the proportion of gut-associated bacteria were higher than that of pelagic fish (such as Desulfovibrionales and Enterobacterales). Therefore, fish gut microbes could reflect the differences in vertical structure of fish habitat.

### 4.2 The invasion of environmental microorganisms plays an important role in the assembly fish gut microbial communities

Our study characterized the intestinal microbial communities of various wild fish and their host habitat in Lake Sanjiao. Due to its status as an urban extensive aquaculture lake, the water body has become eutrophic (Xia et al., 2022), resulting in a dominance of Cyanobacteria within the water microbiota. Consistent with other studies on the gut microbiota of freshwater fish, Proteobacteria and Firmicutes were found to be the dominant phyla in their guts (Bi et al., 2021; Duan et al., 2021) (Figure 2A). The sediments microbiota exhibited the highest α-diversity in all sampling groups (Supplementary Figure S2), predominantly composed of Proteobacteria.

The general introducing-then-filtering framework suggests that the microbes of fish gut contents are mainly transient and were originated from environmental microbes by fish feeding (Yang et al., 2022). To investigate how the habitat (water/sediments) affected fish gut microbiota, our analysis on βNTI proved that the composition of gut content microbiota was dominated by stochastic processes (Supplementary Figure S6), which verified the correctness of the introducing-then-filtering framework. However, our results showed that fish gut microbiota performed more similarly to the sediments microbiota than to the water microbiota (Figure 3A).

### 4.3 Habitat preference was the dominant factor that influence the composition of the fish gut microbiota

Understanding and deciphering the ecological succession of fish gut microbiota are helpful to host metabolism, health, and environmental adaptation (Ghanbari et al., 2015; Robinson et al., 2018), and therefore has become a central theme of gut ecology (Xiao et al., 2021). Our study revealed that the fish gut microbiota exhibited significant intra-group coherence and inter-group divergence, which was consistent with the results of previous studies (Bi et al., 2021; Yang et al., 2022). However, unsupervised machine learning results failed to differentiate from fish gut microbiota at the species level, but instead identified an optimal clustering pattern at *k* = 2, suggesting that other factors beyond host species may influence the composition of fish gut microbes (Figure 4A).

Habitat preference was verified to be the dominant factor affecting the composition of fish gut microbes. Supervised and unsupervised machine learning algorithms had been used to assess discriminative structural factors in the gut microbiota (Kim et al., 2021). The results supported the importance of habitat (Figure 4). We found no significant effect of fish trophic level and fish taxonomy on fish gut microbiota. The possible reasons for this phenomenon are all fish live in the same lake and have a relatively uniform diet, reducing the overall dietary variation. On the other hand, fish are unable to forage when they were collected, which results in empty intestinal tracts and subsequently lose most microorganisms associated with their food. Therefore, these trophic level differences may not significantly correlate with transient changes observed in the fish gut microbiota.

Moreover, while the functional profiles predicted by 16S rRNA gene amplicon sequencing in our study can distinguish fish with different habitat preferences in a supporting role, it is important to acknowledge that RNA-Seq method is better for study on functional diversity. We are looking forward to seeing more studies focusing on RNA-Seq for functional analysis of the fish gut microbiome in the future.

### 4.4 Pelagic fish exhibited more stability gut microbial co-occurrence network than benthic fish

Understanding gut microbial interactions and stability is an important but largely ignored ecological issue, which will be able to guide gut microbial management for providing better ecological services (De Schryver and Vadstein, 2014; Xiao et al., 2022). An interesting phenomenon was found that pelagic fish had a more robust gut structure by the co-occurrence network analysis (Figures 5C,D), which can be explained by the combination of the habitat preferences and the characteristics of the water and sediments. In the water column of the lake, microbial composition appeared to be homogeneous so that little turnover happened in the gut microbiota of pelagic fish. However, heterogeneity horizontally existed in the sedimental microbial composition (Zhai et al., 2022), which gave rise to the high turnover in benthic feeding habitat fish gut microbiota. Therefore, continuous interaction between the highly heterogeneous sediments and fish led to a high frequency of exchanges in fish gut contents, resulting in a decrease in the relative abundance of resident bacteria in the gut microbiota of the benthic fish. Finally, the robustness of the benthic fish gut microbial network was reduced.

## 5 Conclusion

This study presented a comprehensive view of the microbial ecology of fish and provided a completely new perspective on the vertical structure of fish habitats in shallow lakes. In particular, our results show that the preferences of fish in the vertical structure of habitat was the main factor affecting their gut microbiota, even beyond the taxonomic level and trophic level of fish. The vertical structure of fish habitat also affected the composition, function, and network stability of fish gut microbes.

## Data availability statement

The original contributions presented in the study are included in the article/Supplementary material, further inquiries can be directed to the corresponding authors.

## Ethics statement

The animal study was approved by Department of Agriculture and Rural Affairs of Hubei Province. The study was conducted in accordance with the local legislation and institutional requirements.

## Author contributions

BZ: Writing – review & editing, Writing – original draft, Visualization, Software, Project administration, Methodology, Investigation, Formal analysis, Data curation, Conceptualization. JX: Writing – review & editing, Visualization, Software, Investigation, Data curation. HL: Writing – review & editing, Supervision, Project administration, Funding acquisition. DZ: Writing – review & editing, Methodology, Investigation, Conceptualization. YW: Writing – review & editing, Methodology, Conceptualization. SL: Writing – original draft, Resources, Methodology, Investigation. FX: Writing – review & editing, Supervision, Investigation, Funding acquisition. MX: Writing – review & editing, Supervision, Funding acquisition.
