# Supplementary material for: Vertical habitat preferences shape the fish gut microbiota in a shallow lake
Source: Front Microbiol. 2024 Mar 18;15:1341303. doi: 10.3389/fmicb.2024.1341303 (PMC10987288; doi:10.3389/fmicb.2024.1341303)
Supplement: Supplementary file 1 [file Table_1.DOCX]

|  | S1 | S3 | S5 |
| --- | --- | --- | --- |
| H(m) | 1.13 | 1.65 | 1.55 |
| SD(m) | 0.72 | 0.67 | 0.55 |
| WT(℃) | 19.8 | 17.7 | 18.6 |
| pH | 8.34 | 7.95 | 8.21 |
| Cond. | 309.8 | 326.7 | 325.1 |
| DO(mg/L) | 9.05 | 8.37 | 9.14 |
| TN(mg/L) | 2.00 | 1.95 | 1.95 |
| NH4-N(mg/L) | 0.71 | 0.73 | 0.41 |
| NO3-N(mg/L) | 0.47 | 0.76 | 0.44 |
| TP(mg/L) | 0.075 | 0.06 | 0.075 |

**Table S1** **Physical and chemical property of water column at sampling sites of Lake Sanjiao.**

**Table S2 Fish species, full length, body length, weight, and group in Lake Sanjiao.**

| Sample ID | Species | Full length (mm) | Body length (mm) | Weight (g) | Habitat Preference | PAM |
| --- | --- | --- | --- | --- | --- | --- |
| AN01 | *Aristichthys nobilis* | 213 | 172 | 105.93 | Benthic | Cluster 1 |
| AN02 | *Aristichthys nobilis* | 210 | 172 | 102.72 | Benthic | Cluster 1 |
| AN03 | *Aristichthys nobilis* | 205 | 165 | 97.23 | Benthic | Cluster 2 |
| AN04 | *Aristichthys nobilis* | 205 | 167 | 99.08 | Benthic | Cluster 1 |
| CA01 | *Carassius auratus* | 215 | 176 | 162.96 | Benthic | Cluster 1 |
| CA02 | *Carassius auratus* | 225 | 185 | 159.85 | Benthic | Cluster 1 |
| CA03 | *Carassius auratus* | 216 | 180 | 158.55 | Benthic | Cluster 1 |
| CA04 | *Carassius auratus* | 210 | 169 | 144 | Benthic | Cluster 2 |
| CB01 | *Coilia brachygnathus* | 232 | 212 | 36.4 | Pelagic | Cluster 2 |
| CB02 | *Coilia brachygnathus* | 223 | 203 | 34.7 | Pelagic | Cluster 2 |
| CB03 | *Coilia brachygnathus* | 231 | 207 | 36 | Pelagic | Cluster 1 |
| CB04 | *Coilia brachygnathus* | 237 | 214 | 38.3 | Pelagic | Cluster 2 |
| CD01 | *Culter dabryi* | 147 | 118 | 22.33 | Benthic | Cluster 1 |
| CD02 | *Culter dabryi* | 160 | 127 | 26.75 | Benthic | Cluster 1 |
| CD03 | *Culter dabryi* | 155 | 125 | 27.23 | Benthic | Cluster 1 |
| CD04 | *Culter dabryi* | 150 | 120 | 24.32 | Benthic | Cluster 1 |
| HL01 | *Hemiculter leucisculus* | 174 | 144 | 43.05 | Benthic | Cluster 1 |
| HL02 | *Hemiculter leucisculus* | 180 | 150 | 44.23 | Pelagic | Cluster 2 |
| HL03 | *Hemiculter leucisculus* | 190 | 160 | 59.62 | Pelagic | Cluster 2 |
| HL04 | *Hemiculter leucisculus* | 195 | 164 | 59.31 | Pelagic | Cluster 2 |
| PS01 | *Pseudobrama simoni* | 128 | 108 | 20.78 | Pelagic | Cluster 2 |
| PS02 | *Pseudobrama simoni* | 145 | 120 | 30 | Pelagic | Cluster 2 |
| PS03 | *Pseudobrama simoni* | 123 | 100 | 19.52 | Pelagic | Cluster 1 |
| PS04 | *Pseudobrama simoni* | 125 | 103 | 18.77 | Pelagic | Cluster 2 |
| TS01 | *Toxabramis swinhonis* | 143 | 115 | 18.9 | Benthic | Cluster 1 |
| TS02 | *Toxabramis swinhonis* | 149 | 117 | 17.3 | Benthic | Cluster 1 |
| TS03 | *Toxabramis swinhonis* | 145 | 116 | 20.5 | Pelagic | Cluster 2 |
| TS04 | *Toxabramis swinhonis* | 145 | 116 | 16.8 | Pelagic | Cluster 1 |

| Sample | Species | N | C | N sted | C sted | Trophci Level |
| --- | --- | --- | --- | --- | --- | --- |
| CD01-04 | ***Coilia brachygnathus*** | 16.41 | -26.47 | 0.19 | 0.63 | 3.731 |
| CA01-04 | ***Carassius auratus*** | 13.96 | -26.46 | 1.34 | 0.35 | 3.012 |
| TS01-04 | ***Toxabramis swinhonis*** | 13.76 | -27.07 | 0.48 | 0.37 | 2.951 |
| HL01-04 | ***Hemiculter leucisculus*** | 13.30 | -26.03 | 0.49 | 0.72 | 2.817 |
| CD01-04 | ***Culter dabryi*** | 12.80 | -26.83 | 0.98 | 1.01 | 2.671 |
| PS01-04 | ***Pseudobrama simoni*** | 12.00 | -27.04 | 0.70 | 0.78 | 2.436 |
| AN01-04 | ***Aristichthys nobilis*** | 10.04 | -25.21 | 0.31 | 0.09 | 1.859 |
| Reference organism | ***Bellamya aeruginosa*** | 10.52 | -25.73 | 0.10 | 0.61 | λ = 2 |

**Table S3 Stable isotope measurements of δ15N in fish. The reference organism is *Bellamya aeruginosa*, and the red line indicates the demarcation of fish by a threshold of 2.8 trophic level, above 2.8 for high trophic level and below 2.8 for low trophic level.** **∆δ^15^N =** 3.4‰.

**Table S4** **The global properties of the whole network based on the relative abundance**

**of ASV.**

| Global properties | benthic | pelagic | sediments | water |
| --- | --- | --- | --- | --- |
| Average Shortest Path Length | 2.76759563 | 2.72868852 | 2.53251366 | 2.6920765 |
| Betweenness Centrality | 0.02850054 | 0.04517391 | 0.08376665 | 0.07087569 |
| Closeness Centrality | 0.36176117 | 0.36672905 | 0.39487258 | 0.37146455 |
| Degree | 123.266667 | 147 | 266.666667 | 169.333333 |
| Eccentricity | 3 | 3 | 3 | 3 |
| EdgeCount | 123.266667 | 147 | 266.666667 | 169.333333 |
| Indegree | 123.266667 | 147 | 266.666667 | 169.333333 |
| Neighborhood Connectivity | 11.1865716 | 9.26875544 | 6.01769786 | 4.95500894 |
| Number Of Undirected Edges | 123.266667 | 147 | 266.666667 | 169.333333 |
| Radiality | 0.99357238 | 0.99371386 | 0.99442722 | 0.99384699 |
| Stress | 12349502 | 16192957.1 | 31091218.7 | 14488264 |
| Topological Coefficient | 0.27531275 | 0.22347988 | 0.13561346 | 0.10689213 |

**Table S5 The global properties of both benthic and pelagic fish gut microbiota co-occurrence networks based on the spearman coefficient.**

| Global properties | benthic | pelagic |
| --- | --- | --- |
| Total nodes | 561 | 667 |
| Total links | 6273 | 5078 |
| R square of power-law | 0.33940058 | 0.42400659 |
| Average degree (avgK) | 22.3636364 | 15.2263868 |
| Average clustering coefficient (avgCC) | 0.75770141 | 0.7372797 |
| Average path distance (GD) | 4.1366687 | 9.7626088 |
| Geodesic efficiency (E) | 0.43824567 | 0.24382996 |
| Harmonic geodesic distance (HD) | 2.28182517 | 4.10121867 |
| Maximal degree | 79 | 44 |
| Centralization of degree (CD) | 0.10113636 | 0.04320362 |
| Maximal betweenness | 6914.60524 | 19036 |
| Centralization of betweenness (CB) | 0.04337623 | 0.08353185 |
| Maximal stress centrality | 17178218 | 985450802 |
| Centralization of stress centrality (CS) | 108.075984 | 4319.98442 |
| Centralization of eigenvector centrality (CE) | 0.869697 | 0.93533835 |
| Maximal closeness centrality | 5.02E-06 | 3.85E-06 |
| Centralization of closeness centrality (CCL) | 0.00122662 | 0.0011931 |
| Density (D) | 0.03993507 | 0.02286244 |
| Transitivity (Trans) | 0.91513519 | 0.97200423 |
| Connectedness (Con) | 0.15651261 | 0.19397959 |
| Efficiency | 0.75272839 | 0.88853302 |

**
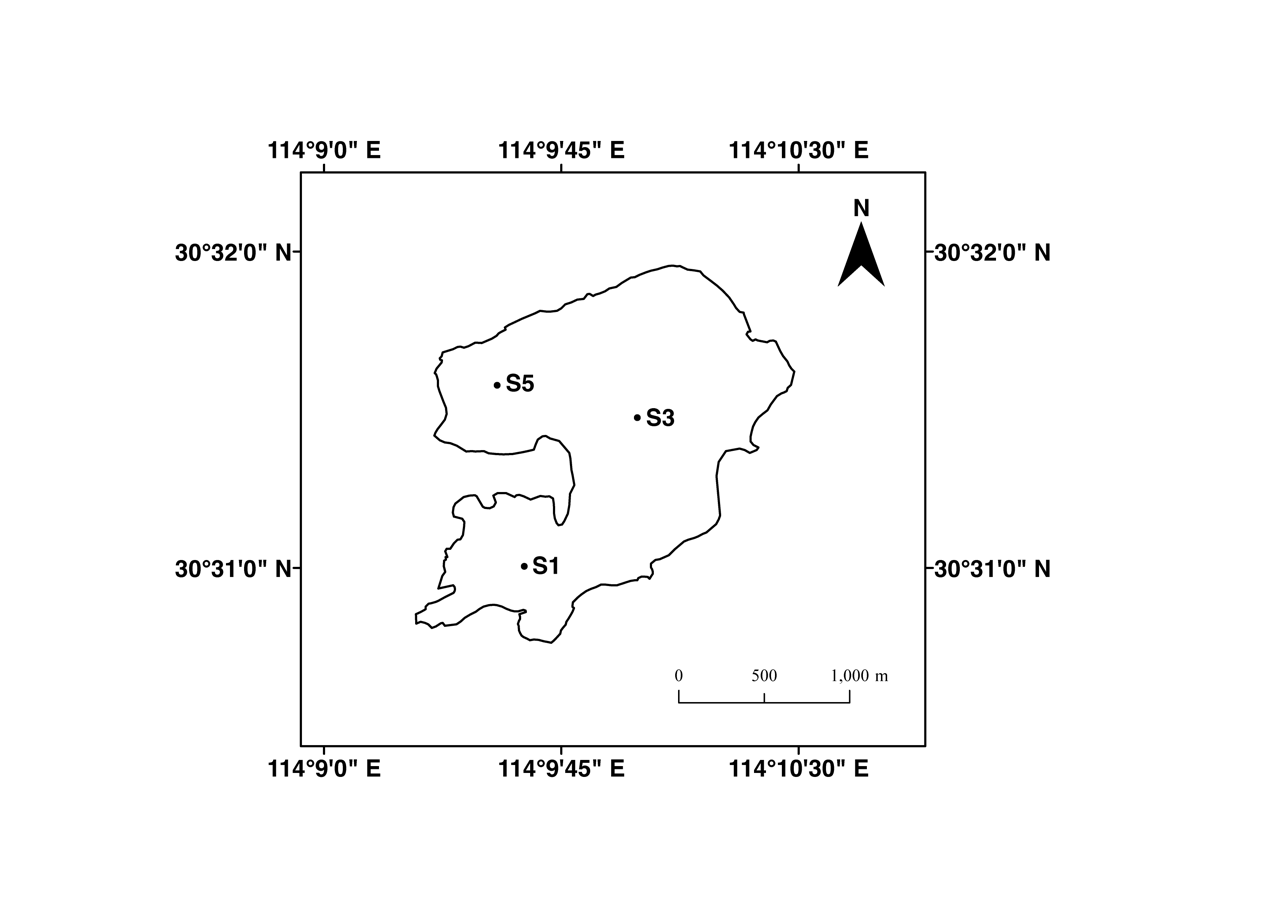
**

**Fig. S1. Sampling sites in Lake Sanjiao.** Water, sediments, and fish were collected at 3 sampling sites.

**
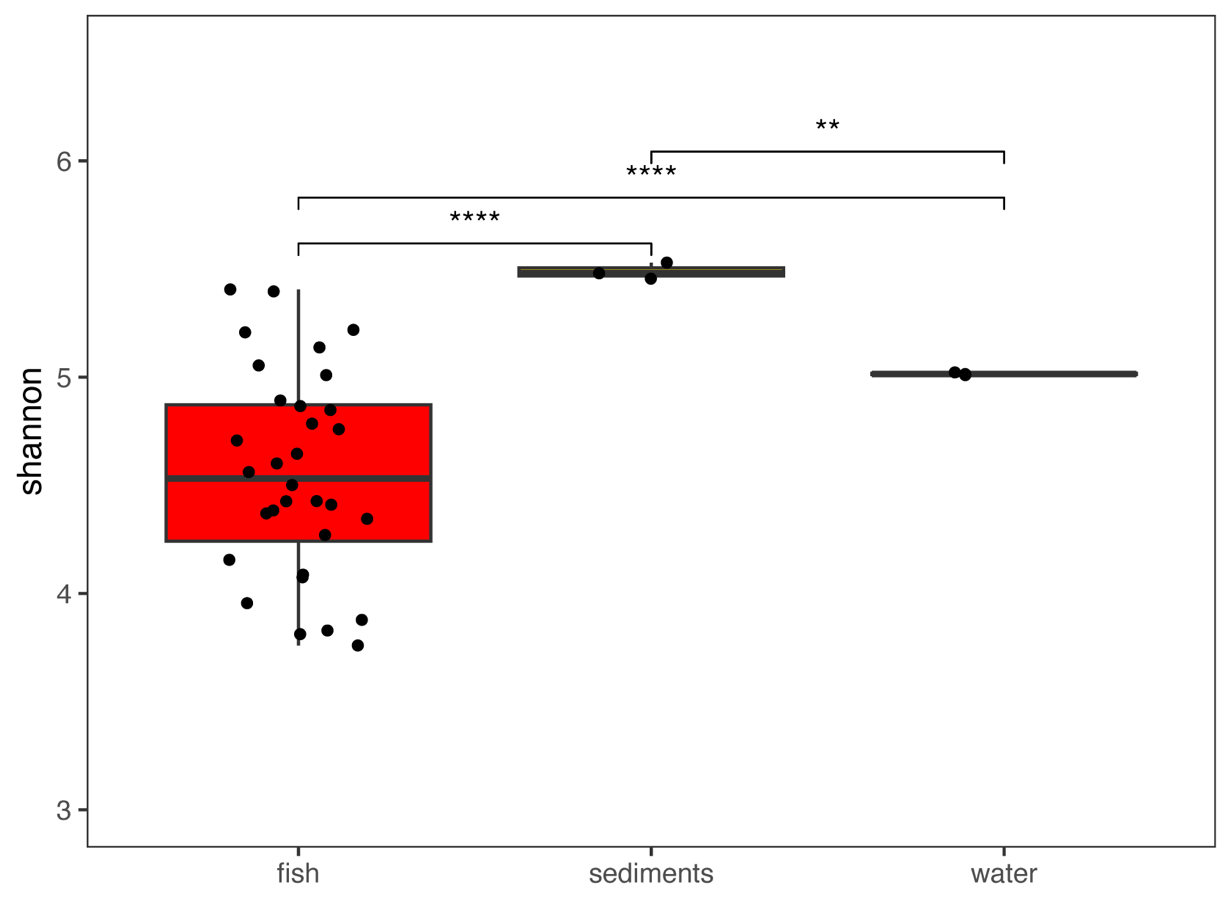
**

**Fig. S2.** Shannon index was brought to evaluated theαdiversity of the water microbiota, sediment microbiota and fish gut microbiota, asterisks indicate t-test significance.

**
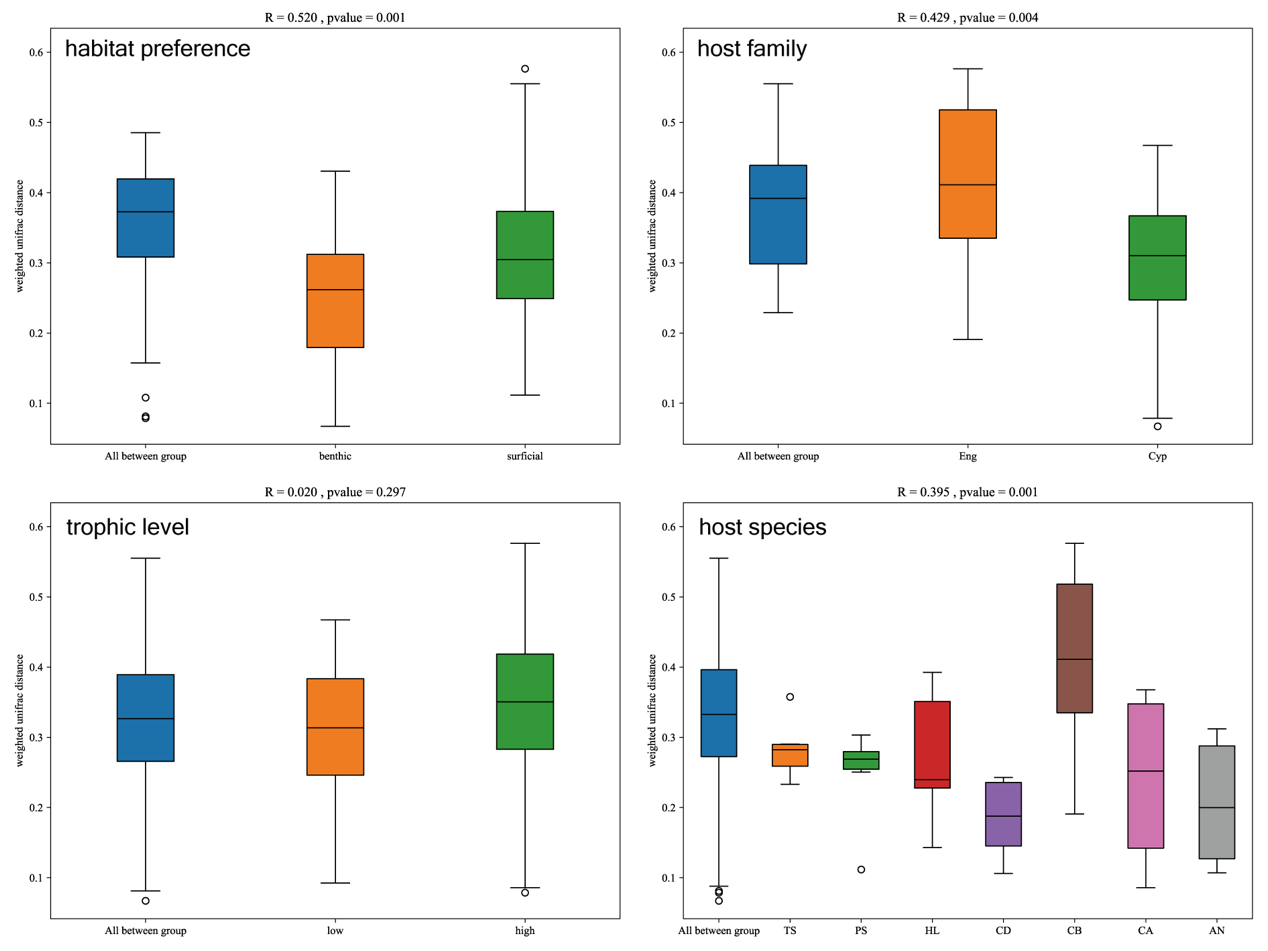
**

**Fig. S3.** Analysis was conducted to determine the impact of habitat preference, fish taxonomy and fish trophic level on fish gut microbiota. The variation was measured using weighted unifrac distances between samples, while ANOSIM with the R "vegan" package was used to calculate size effect and statistical significance.

**
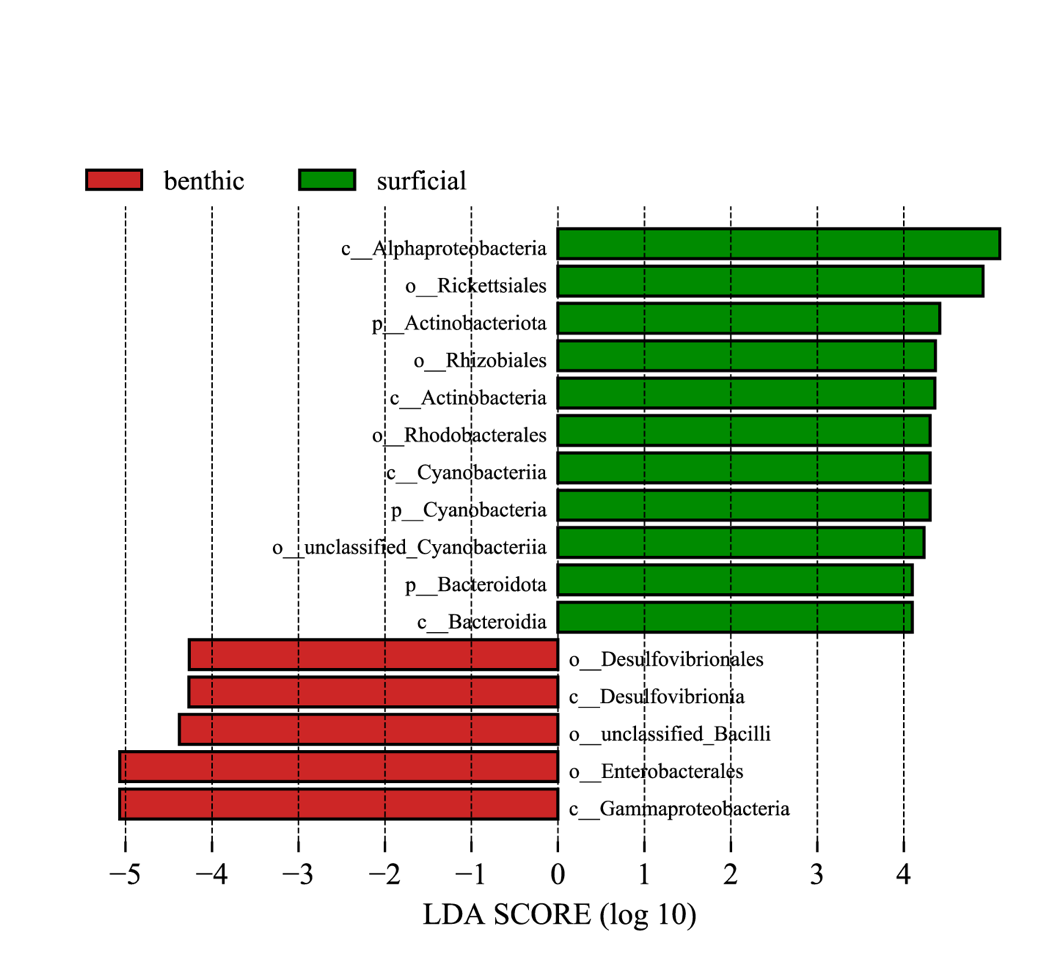
**

**Fig. S4.** **Most discriminative bacterial taxa between the gut microbiota of pelagic and benthic fish.** Bacterial that differ significantly in abundance between benthic fish and pelagic fish, as analyzed by LEfSe (LDA score > 4).

**
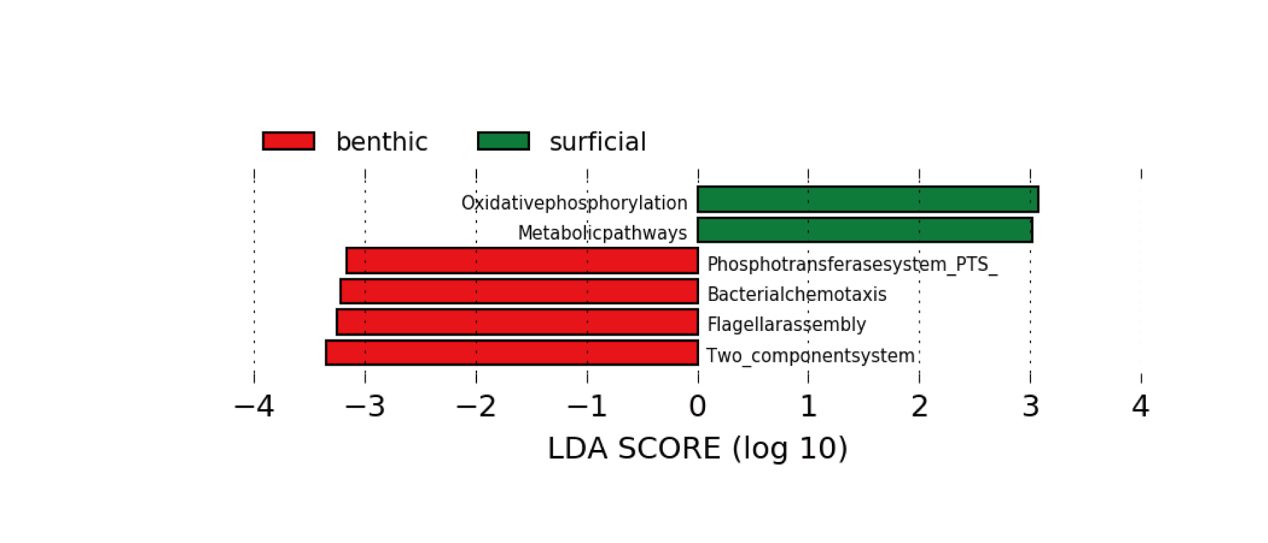
**

**Fig. S5. Most discriminative function profile between the gut microbiota of pelagic and benthic fish.** Functions that differ significantly in abundance between benthic fish and pelagic fish, as analyzed by LEfSe (LDA score > 3).


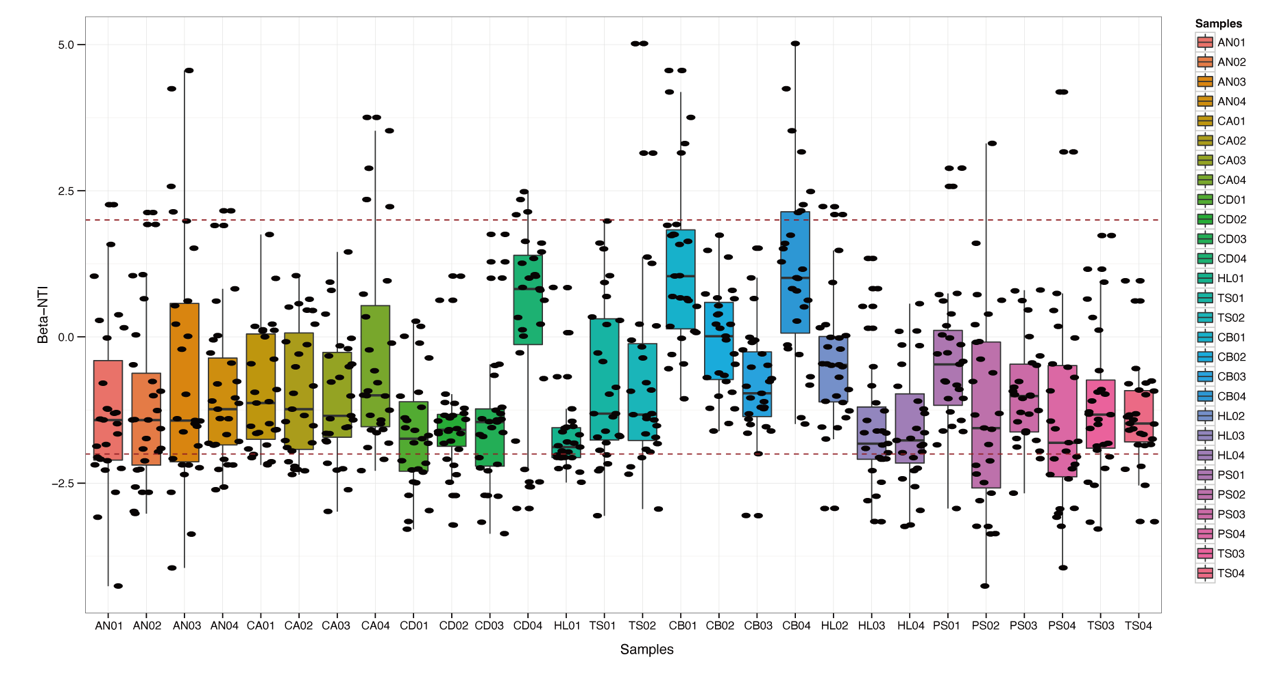


**Fig. S6. The distribution of β-NTI values in 28 fish gut microbiota.** The βNTI values were concentrated in the range of -2~2, indicating that the community assemblies were mainly dominated by stochastic processes.
